# Veterinary Medical Association of New Jersey

**Published:** 1901-02

**Authors:** George W. Pope

**Affiliations:** Secretary


### VETERINARY MEDICAL ASSOCIATION OF NEW JERSEY.

Had any veterinarian in the State of New Jersey an impression that the amalgamation of the three State societies, which was accomplished just one year ago at the meeting in Newark, was not completed and that the veterinary forces of the State were not united in a loyal and unselfish purpose, attendance at the meeting held at Trenton on January 10th would have banished from his mind all doubt or question. Although the day was dark and stormy, in fact, one of the most disagreeable of the year, the attendance and spirit of the meeting was most gratifying, the number present being upward of forty and the fraternal feeling exhibited by every man such as to be long remembered. Another indication of prosperity was the application of twelve men for membership. Messages were received from four members who were detained at home on account of sickness or death of friends or relatives. Among this number was the President, Dr. Hurley, of Hopewell, and in his absence the First Vice-President called the meeting to order.

The principal business of the day was the report of the Committee on Revision of the Constitution, By-Laws, and Code of Ethics. The committee presented a revised Constitution. The Board of Censors recommended the same with a few changes. The universal feeling of the meeting was that the adoption of a Constitution for the government of an organization was a matter of extreme importance, and accordingly each article was taken up separately and carefully considered, the greater portion of the session being devoted to the settlement of questions which arose relative to the advisability of certain changes. All questions were discussed in a most kindly manner, and the revised Constitution was finally adopted, to the satisfaction of all present.

Following the adoption of the Constitution occurred the election of officers for the ensuing two years.

Dr. T. Earle Budd, of Orange, in the following well-chosen words, nominated Dr. William Herbert Lowe, of Paterson, for President.

*Nominating Speech.*

Mr. President and Gentlemen: Our attendance to-day is an expression of our loyalty to our former societies and a belief that this Society in the future will exert a wide influence and will foster in each member a desire to regularly attend its meetings and by thought and interest put this Society and the profession itself in the rank to which it belongs—second to none.

In presenting the name of one who will be the President of our New Jersey Veterinary Society for the coming two years I shall present one to you who has been weighed in the interests of our Society and the veterinary profession and not found "wanting." I shall present one who has held positions of trust in our government for eight years, and when his place was filled by others he left a record that his successor would be willing and glad to follow and the veterinary profession proud to point to. He now stands as the Treasurer of our American Veterinary Medical Association.

It is also his untiring zeal and unremitting efforts which have taken our weak and ineffective societies and merged them into a body that is to be known as the New Jersey Veterinary Medical Association—a Society which will be strong in its work in advancing our profession and a help and stimulus to each of its members.

It is also to his efforts that we are to be honored in having the American Veterinary Medical Association meet in our State next September.

Gentlemen, it is, I think, an honor that to-day, in this the beginning of our new century, we have the privilege of making Dr. William Herbert Lowe our President, whose name I bring before you.

Personally, I feel it an honor to present the name of William Herbert Lowe, of Paterson, as the President of the New Jersey Veterinary Medical Association for the years 1901 and 1902.

Dr. Lowe was elected by a unanimous vote, was escorted to the Chair, and delivered the following inaugural address, which we wish might be read by every veterinarian in our land.

*President Lowe's Inaugural Address.*

FELLOW-MEMBERS: I rejoice in the complete unification of all factions of the profession in this State, harmoniously and triumphantly accomplished during the closing year of the old century.

With the obliteration of all factional differences, and with a common interest and a common devotion to a common cause, the possibilities and prospects for advancement along broad and advanced lines in our science and art are almost illimitable. Like every great movement, there had to be some individual sacrifices made; but these sacrifices, made for the common good of the profession, have strengthened the ties of friendship and mutual respect such as nothing else could do.

By your voice, and with your vote, you have seen fit this day to confer upon me the unique and distinguished twofold honor of being the first President-elect of the Amalgamated Association of the State of New Jersey as well as the first President of the new century.

This is, indeed, an honor that any man in the profession might feel proud of; but the thought that is uppermost in my mind at the present time is that the responsibilities and opportunities of the executive officer of the Veterinary Medical Association of New Jersey, as now constituted, are infinitely greater than has ever before fallen to the lot of any veterinarian in our State.

The progress of this Association must ever be along scientific and broad lines, with a constant view to applicability and practical usefulness. Nothing short of this will satisfy the veterinary practitioner of the twentieth century. How well this shall be accomplished in New Jersey depends largely upon you. Your President will outline and direct various lines of investigation and organized work, but the success of his administration will depend, in a large measure, upon the earnest and loyal support that each member gives him in his official capacity. Let each member do his part well, whether it be much or whether it be little, and the result will be simply marvellous.

Less time must be spent in the transaction of routine business and more time devoted to the consideration of the scientific and practical problems of our profession. Busy practitioners will not leave their practice and come to our meetings unless they profit by so doing. We must make the meetings so interesting and instructive that practitioners cannot afford to be absent; then the Association will be a success.

Another thing—be broad and liberal-minded. Things will occur at our meetings that you, individually, do not approve of. Do not get disgusted and stay away from the next meeting, but, on the contrary, be sure to come and do your part to correct what you believe to be wrong.

Then, again, do not stay away from meetings of the State Association because there are some members that you do not like or that you do not consider well qualified. You can associate with those who are congenial, but, above all, remember that those who are less competent than you need the benefit of the organization more than those who are better qualified.

Two very important committees are created by the new By-Laws adopted to-day. The breadth, scope, and practical value of the work of these committees to the public are, so far as I know, in advance of that of any similar committees anywhere in existence.

It is made the duty of the Public Health Committee to investigate, advise, and report on animal diseases, animal foods, sanitation, and other matters relating to and concerning the public health. The importance and value to mankind of the work of this committee alone, if intelligently conducted, cannot be estimated.

The other important committee that I refer to is to be known as the Animal Industry Committee, which shall investigate, study, and report on all practical questions relating to the breeding, maintenance, and utilization of animals, with a view of fostering and placing animal husbandry, in all its phases, upon a more scientific, economic, and profitable basis in this State. Many Associations have committees on diseases, and on contagious diseases, etc., but sight has been lost, in many instances, at least, of the practical value of a scientific application of the physiological laws governing the animal industry. This phase of this important subject has been left almost entirely to the layman to manage the best he could. No wonder the public should have a restricted idea of the veterinarian, and no wonder that a newspaper reporter should say that he did not think an account of the meeting would interest the general public. I agree with him that the routine proceedings would not interest the general public, but I know, at the same time, that if the Association was doing the work that properly comes within its scope there would be much that the newspapers would be looking for. We as a profession are not in close enough touch with agricultural, livestock, dairy, public health, sanitary, and kindred associations and boards. Our members should meet with organizations of this class and their members should be encouraged to meet with us.

The establishment of these committees inaugurate two inexhaustive fields for the progressive veterinarian. There will be plenty of work in the future for many veterinarians in connection with breeding, maintenance, and utilization of animals, even if there should be no sick ones to treat. Then, again, there remains for the veterinarian much to be done in preventive medicine and sanitary science that will be a boon to the human family.

Gentlemen, I thank you.

Other officers elected were: First Vice-President, Dr. T. Earle Budd, of Orange; Second Vice-President, Dr. J. O. George, of Camden; Secretary, Dr. George W. Pope, of Garfield; Treasurer, Dr. B. F. King, of Little Silver.

A vote of thanks was extended to the retiring officers who had borne the heat and burden of the day. Special mention is here made of Dr. Lockwood, who retired from the secretaryship after a faithful service of seven years.

Dr. W. Horace Hoskins, of Philadelphia, made a flying trip to Trenton in the afternoon, and by his presence and a few timely words of cheer and encouragement added to the inspiration of the day.

At 6 P.M. the meeting adjourned, to meet in Newark in July.

GEORGE W. POPE, V.S.,
Secretary.

